# Protection of farm goats by combinations of recombinant peptides and formalin inactivated spores from a lethal *Bacillus anthracis* challenge under field conditions

**DOI:** 10.1186/s12917-017-1140-2

**Published:** 2017-07-12

**Authors:** Susanne M. Koehler, Fatih Buyuk, Ozgur Celebi, Hayati Demiraslan, Mehmet Doganay, Mitat Sahin, Jens Moehring, Okechukwu C. Ndumnego, Salih Otlu, Henriette van Heerden, Wolfgang Beyer

**Affiliations:** 1 Department of Infectiology and Animal Hygiene, University of Hohenheim, Institute of Animal Science, 70593 Stuttgart, Germany; 2 Faculty of Veterinary Medicine, Department of Microbiology, Kafkas University, 36300 Kars, Turkey; 3 Faculty of Medicine, Department of Infectious Diseases, Erciyes University, 38039 Kayseri, Turkey; 4 Institute for Crop Science, University of Hohenheim, Biostatistical Unit, 70593 Stuttgart, Germany; 5 Department of Veterinary Tropical Diseases, University of Pretoria, Onderstepoort, 0110 South Africa; 6 Robert-Koch-Institut, 13353 Berlin, Germany; 7 Africa Health Research Institute, Durban, 4013 South Africa

**Keywords:** Bacillus anthracis, Anthrax, Vaccination, Animal vaccine

## Abstract

**Background:**

*Bacillus* (*B.*) *anthracis,* the causal agent of anthrax, is effectively controlled by the Sterne live spore vaccine (34F2) in animals. However, live spore vaccines are not suitable for simultaneous vaccination and antibiotic treatment of animals being at risk of infection in an outbreak situation. Non-living vaccines could close this gap.

**Results:**

In this study a combination of recombinant protective antigen and recombinant *Bacillus* collagen-like antigen (rBclA) with or without formalin inactivated spores (FIS), targeted at raising an immune response against both the toxins and the spore of *B. anthracis*, was tested for immunogenicity and protectiveness in goats. Two groups of goats received from local farmers of the Kars region of Turkey were immunized thrice in three weeks intervals and challenged together with non-vaccinated controls with virulent *B. anthracis,* four weeks after last immunization. In spite of low or none measurable toxin neutralizing antibodies and a surprisingly low immune response to the rBclA, 80% of the goats receiving the complete vaccine were protected against a lethal challenge. Moreover, the course of antibody responses indicates that a two-step vaccination schedule could be sufficient for protection.

**Conclusion:**

The combination of recombinant protein antigens and FIS induces a protective immune response in goats. The non-living nature of this vaccine would allow for a concomitant antibiotic treatment and vaccination procedure. Further studies should clarify how this vaccine candidate performs in a post infection scenario controlled by antibiotics.

**Electronic supplementary material:**

The online version of this article (doi:10.1186/s12917-017-1140-2) contains supplementary material, which is available to authorized users.

## Background

Anthrax was one of the first bacterial diseases to be controlled by vaccination. Starting with the famous vaccination of sheep conducted at Pouilly-le-Fort, France, by Louis Pasteur [1] his duplex vaccine became widely used for cattle and sheep in Europe and South America for about 50 years. It was, however, Max Sterne’s live-spore vaccine [2] which in the long run became the world’s most used live vaccine against anthrax in the veterinary field [3]. It remains the veterinary vaccine in use in most countries encountering anthrax [4].

Sterne’s own trials with his vaccine [5, 6] had shown effectiveness in diminishing outbreaks in South Africa over the period 1925 to 1941. Nevertheless, while proving effective in controlling anthrax worldwide over the last 75 years, usage of the Sterne live spore vaccine poses a number of problems. These include problems related to the standardization of vaccine production and avoidance of batch to batch variation in content and immunogenicity, and the residual virulence known from several laboratory species and livestock [2, 7–9], in particular goats [3]. More importantly, especially for use in livestock and endangered wild species, the live vaccine has been noted to prevent development of vaccine induced antibodies when administered concomitantly with an antibiotic in small laboratory rodents [10, 11]. The simultaneous antibiotic treatment and vaccination would be of great importance for control of an outbreak situation where the possible infection of livestock or wild animals needs to be prevented after the activity of the antibiotic has ceased. This concept has led to the approval of a human vaccine (AVA, (BioThrax^R^), Emergent BioSolutions) for a post-exposure scenario in humans where antibiotics may be administered for up to 60 days [12]. In animals, the risk for infections by spores originally present in or newly shed to the environment by early victims of an outbreak is even much higher, as one-shot treatments with long-lasting antibiotics generally do not protect for more than 14 days.

So far non-living alternatives are not available for veterinary use, contrary to the licensed acellular human vaccines AVA and AVP whose main immunological ingredient is the so-called protective antigen (PA; see reviews in [13–16]). However these are imposed with limitations of their own like an extensive immunization schedule, batch to batch variations in the content and concentration of the antigens [17], and species-specific variations in the protective capacity [18–22]. Thus the search for more defined recombinant protein antigens analogous to the licensed acellular human vaccines is still ongoing.

Varying results have been published in laboratory animal models (reviewed in 13) with purified PA preparations and recombinant PA (rPA). Results indicated that vaccines solely based on the toxin derived antigens, mainly PA, are less protective than live-spore vaccines against virulent isolates of *B. anthracis* [7, 23–29]. Spore vaccines constructed from toxigenic, non-encapsulated strains have repeatedly demonstrated the capacity to confer protection in mice and guinea pigs against challenge with virulent *B. anthracis* [7, 9, 23, 24, 30, 31]. Better protection by live spore vaccines compared to AVA or AVP has been attributed to improved stimulation of the cellular or cell mediated immune response by both spore and vegetative cell components [7, 8, 23, 32].

Brahmbhatt et al. [33] showed a protective effect of an exosporium derived antigen, *Bacillus* collagen-like antigen (BclA), when added to suboptimal amounts of PA in a combination vaccine. This supported the results of Hahn et al. [34] that showed the protection afforded by a combination of rPA and rBclA encoding plasmids was significantly better than protection provided by vaccination with the single components. More recently it was shown that other spore derived antigens [27, 29, 35–37], surface layer proteins [28, 38] or inactivated full spore preparation such as FIS [39–41] can add to the protective efficacy of PA-based vaccines.

The PA-induced immune response is highly dependent on the adjuvant used [19, 20]. In recent trials a lipopeptide adjuvant (LP), already validated in other contexts [42–47], was compared with alhydrogel (Ah) in mice and rabbits. Animals immunized with rPA and LP had significantly higher antibody (IgG2a) titers than those given rPA and Ah. When NMRI mice were challenged subcutaneously with *B. anthracis* Ames spores the rPA/LP immunized group survived significantly better than both the negative controls and the rPA/Ah immunized group, whereas rabbits were completely protected. None of the animals showed clinical side effects with this adjuvant [48].

As early as in 1946 Gladstone [49] recognized the potential of a non-cellular vaccine against anthrax and tested a culture filtrate of *B. anthracis* in several laboratory species and sheep. Experiments in the 1950s in cattle, sheep, and pigs demonstrated the potential protectiveness of such a cell-free vaccine [50, 51], though the interpretation of these trials was always hampered by the inability to find a fully lethal dose with the challenge strains used. To the best of our knowledge there have been no further studies aimed at protecting livestock animals from a virulent infection with *B. anthracis* by a non-living vaccine since these early experiments.

Our study investigated the protective immune response induced by a combination of recombinant proteins with or without FIS in goats. This species was selected given the extremely high susceptibility of goats and their economic and social importance in many arid or semi-arid areas of the world. A threefold vaccination with a non-living vaccine comprising of rPA, rBclA and FIS as the relevant antigens in combination with LP adjuvant revealed 80% survival after a parenteral lethal challenge with spores of *B. anthracis*. This indicates the high potential of such vaccines as either a supplement or even alternative to living spore vaccines when (i) vaccination is desirable to prevent the re-activation of an outbreak after the initial antibiotic treatment or (ii) when other non-living components are used in so-called combination vaccines to immunize simultaneously against both *B. anthracis* and a different pathogen. Such combination vaccines are often used concomitantly with antibiotics in the veterinary practice.

## Methods

### Animals

Goats were purchased from local farmers and comprised of a phenotypically heterogeneous group of both males and females of ages around 1 year, belonging to the breed of Caucasian goats. Animals were sourced from farms not affected by anthrax and with no anthrax vaccine history in the last 5 years. All animals were tested for anti-PA antibodies by ELISA and only animals considered negative were enrolled. Initially 25 goats were held in one flock on a fenced pasture throughout the vaccination. To allow for acclimatization the goats were held at the site of the experiment for approximately 1 month prior to the start of the vaccination. All animals were treated with Ivermectin (7 mg, s. c.) and mineral supplement (Depomin, 10 ml orally per day, for 3 days) and the health status was monitored by local veterinarians. Two vaccine groups of 10 goats each (groups A and B) and one unvaccinated negative control of 5 goats (group NegCTL) were selected at random from the flock to make up the experimental groups. During the challenge period groups of animals were held in separate concrete barns equipped with water basins and racks containing hay. Hay was provided ad libitum. At the end of the trials barns and any non-burnable materials were disinfected by spraying with 10% formaldehyde solution. Carcasses and contaminated materials were incinerated. Two animals of group A and one animal in group NegCTL were lost during the time of vaccination but before challenge (Table 1) due to reasons unrelated to the vaccination procedure (two predations and one unknown loss). The immunological data of these animals were part of the evaluation.

### Recombinant proteins

*Escherichia coli* BL21-CodonPlus-RIL cells (Stratagene, La Jolla, CA) harboring the plasmid pREP 4 and pQE-30 (both Qiagen, Venlo, Netherland) encoding either rPA83 or rBclA coupled to a HIS-tag were grown and purified as described previously [34]. Briefly, bacterial inoccula were grown in antibiotic supplemented Standard medium I (Roth, Karlsruhe, Germany). Upon reaching OD_600nm_ of 0.6, protein production was induced with IPTG (Roth, Karlsruhe, Germany). After incubation for 3 h at 37 °C shaking cultures were lysed and proteins purified on a HiTrap Sepharose column loaded with nickel(II)sulfate (Sigma-Aldrich, Taufkirchen, Germany) via FPLC with Äktaprime plus (GE Healthcare, Freiburg, Germany). After dialysis onto 5 mM HEPES-buffer (Roth, Karlsruhe, Germany) and quality assessment via SDS-page, protein yield was established via Bicinchoninsäure test (Interchim, Montlucon, France). Proteins used for ELISA received no further treatment while proteins used for vaccination were tested for endotoxin (ET) yield using the Limulus Amoebocyte Lysate Endochrome-K test kit (Charles River, Wilmington, MA) according to the manufactures’s instructions. Endotoxin removal was performed via EndoTrap blue endotoxin removal system (Hyglos, Bernried, Germany) for proteins with ET values more than 50 ng/kg body weight.

### Formalin inactivated spores (FIS)

Sterne vaccine strain 34F2 spores (strain collection of the University of Hohenheim) suspended in 0.9% NaCl containing 0.1% gelatin were pelleted, then re-suspended in PBS with 4% formalin and incubated at 37 °C overnight. The suspension was centrifuged (4000 x g) four times and re-suspended in 0.9% NaCl and 0.1% gelatin with the last pellet re-suspended in endotoxin free PBS. Sterility of 1 aliquot of the preparation was confirmed on blood agar after neutralization of formalin with 1 volume of a solution containing 20 g/L histidine at room temperature for 30 min.

### Lipopeptide adjuvant

A lipopeptide adjuvant comprising Pam3Cys-SKKKK, a TLR2/1 activator admixed with Pam3Cys conjugated to the promiscuitive T-helper-cell epitope of the sperm whale myoglobin SFISEAIIHVLHSRHPG [46, 47, 52] was purchased from EMC Microcollections GmbH, Germany.

### Serology

All sera were tested individually for IgG antibodies against the respective antigens rPA83, rBclA, and FIS in the ELISA. IgM titers against rBclA and FIS were assessed with pooled group sera from each sampling point. The ELISA tests were performed as described earlier [53]. Briefly 96-well Maxisorp plates (Sigma-Aldrich, Taufkirchen, Germany) were coated with 0.5 μg antigen or respectively 10^8^ cfu FIS per well in coating buffer (8.4 g/L NaHCO_3_, 3.56 g/L Na_2_CO_3_) over night. Sera were titrated on the plate in duplicates using a serial log_2_ dilution with a starting concentration of 1:100. Controls included an equivalently titrated goat serum positive for all antigen tested, as well as point measurements (concentration 1:100) of unvaccinated goat sera as a negative control. The secondary antibody rabbit anti-goat IgG HRP (Thermo Fischer, Waltham, MA USA) was used at a dilution of 1:6000. Endpoint titers were developed with ABTS (Roche, Mannheim, Germany) and defined as the reciprocal of the highest serum dilution that resulted in an OD_414nm_ greater than 0.1.

Toxin-neutralizing assay (TNA) was performed as published earlier [53] to qualitatively assess the ability of antibodies generated to neutralize lethal toxin. Briefly, heat inactivated individual sera were serially diluted in 8 log_2_ steps with a starting dilution of 1:50 on sterile 96 well plates (BD Biosciences, Heidelberg, Germany) seeded with 10^5^ J774 A.1 macrophages per well. Lethal toxin (LF) was added at concentration of 500 ng/mL PA and 100 ng/mL LF (List Biological Laboratories, Campbell, CA, USA) and survival of macrophages was visualized through MTT (5 mg/mL, Alfa Aesar, Karsruhe, Germany), which is degraded by living cells to a measurable (OD540_nm_) colour compound. The neutralization titer (NT) corresponds to the inverse serum dilution at which the macrophage survival yielded 50% (NT_50_). ELISA titers and NT_50_ were estimated using the Sigma Plot regression wizard (4-parameter sigmoid regression curve).

### *Bacillus anthracis* challenge strain

The virulent *B. anthracis* strain was isolated from the spleen of a deceased bovine in the Kars region (strain K-136, collection of the Department of Microbiology, Faculty of Veterinary Medicine of the Kafkas University, Turkey) and identified as described earlier [54]. Spores were prepared as described in the WHO guidelines “Anthrax in humans and animals” [4]. The presence of both virulence plasmids pX01 and pX02 was tested by PCR [4] from 10 single colonies randomly picked from an overnight culture of the spore suspension on blood agar. The virulence of the challenge strain was confirmed in BALB/c mice at Erciyes University Hakan Cetinsaya Animal Experimentation Laboratory, Kayseri/Turkey. For this, two groups of 2 mice were challenged with 625 or respectively 1250 spores given s. c. in the back. Death occurred within 68 h after the lethal challenge and was verified through re-isolation of the challenge strain from heart and lung tissue.

### Immunizations and challenge

**Fig. 1:**
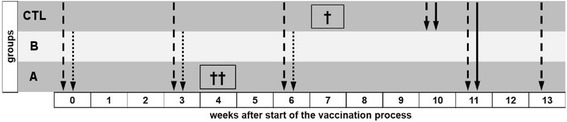
Timeline of the vaccination and challenge schedule. Dashed arrows indicate blood sampling; dotted arrows indicate vaccination time points and continuous arrows indicate the application of the challenge dose; Arrow’s lengths indicate schedule of the relevant groups (A-C); † indicates death of individual goats unrelated to the experimental set up within the indicated time frame; In week 10 only 2 individuals of group NegCTL (unvaccinated negative control) were bled and challenged to verify the functionality of the challenge set up, the remainder of group C was challenged and bled in week 11 together with group A (rBclA + rPA83), and B (rBclA + rPA83 + FIS). FIS: Formalin inactivated spores; rBcLA: Recombinant *Bacillus* collagen-like antigen; rPA: Recombinant protective antigen

Goats of group A received a vaccine formulation comprising of 500 μg LP adjuvant, 75 μg rPA83 and 75 μg rBclA per dose and group B goats received the same formulation as A with the addition of 10^8^ FIS per dose. The 75 μg of recombinant proteins was a 25-fold increase in PA that is present in a single dose of the human vaccine AVA [55]. The spore dose was as published for studies with mice and guinea pigs [39]. Group NegCTL served as an unvaccinated negative control without any injections. The groups were vaccinated three times with 3 week intervals by subcutaneous injection into the upper thigh of the hind leg with 1 mL of vaccine (components diluted in 1 mL of endotoxin free PBS). Blood samples were collected before each immunization, right before and 14 days after challenge from survivors (Fig. 1). All animals were challenged with approximately 1000 spores at one time, except for two randomly chosen members of group NegCTL (96,163 and kesik kulak) that were challenged before the rest of the groups to establish the virulence of the spore suspension prepared. Re-counting colony forming units on blood agar plates revealed a number of 1027 spores for the two negative controls challenged first and 918 spores for the other goats.

To assess the health status of the challenged goats the general behaviour was checked and rectal temperature was measured at least twice daily for 2 weeks after the challenge. In cases of temperatures of ≥40 °C a blood smear to determine the presence of encapsulated bacilli in the blood was made [55]. Animals with negative blood smears were monitored more frequently. Positive animals were treated with 1 mL/25 kg body weight Dipenisol (penicillin/streptomycin formulation, Bayer, Leverkusen). The day of treatment was recorded as “time of death” for the analysis of protectiveness. The animal trials were approved by the Ethic Committee of Animal Experimentation of the Erciyes University, No. 13/01, decision date 09.01.2013.

### Statistics

Titer values for all four traits were transformed using a logarithmic transformation, as residuals visually indicate clearly increasing variances for larger expected values. These data were then analyzed using a mixed model assuming fixed effects for groups and time points and their interactions. Furthermore, the model accounts for repeated measures taken from each goat. Repeated measures can be modelled by an unstructured variance covariance matrix for observations from one goat, but for all traits fitting a random goat effect and a week-specific error variance increased model fit measured by the Akaike information criterion (AIC). Values under the detection limit were replaced by the halved value of the detection level on the transformed scale. If all observations from one time point or one group were under the detection level, the variance for these observations is zero. To avoid underestimation of variances, we dropped these observations and present means for them as under detection level in the results section. A Tukey tests accounting for multiple testing were performed only after finding differences for a factor via F-Test. A letter display was used were means with identical letters were not significant different from each other. Additionally, in case of non-significant interactions between group and time point simple means were calculated, but not used for testing. Predicted means were back transformed for presentation only. Standard errors were back transformed using the delta method. As titer values could only be measured from surviving goats, means from the last time point depend on the surviving of goats. For those titers animal effects were correlated for the traits anti-rPA83 and NT_50_ using a bivariate model. To elaborate survival time the log rank test was employed using data from all treated goats. Multiple comparison was done using the adjustment by Tukey. For all statistical evaluation a *P*-value of 0.05 was considered significant. All analysis were done using the procedures PROC MIXED and PROC LIFETEST of the SAS system.

## Results

### Anti-rPA-IgG titer

**Fig. 2:**
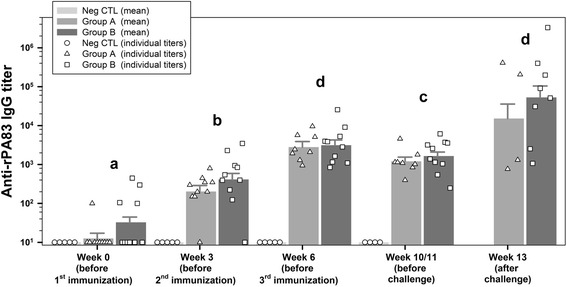
Anti-recombinant PA (rPA) ELISA titers. Displayed are IgG-antibody-values for individual animals as well as predicted means and standard deviations for group A (rBclA + rPA83), B (rBclA + rPA83 + FIS) and NegCTL (unvaccinated negative control) on all sampling points measured via indirect ELISA against rPA83. Values below the detection limit (<100) were given an arbitrary value of 10. Individual values are given as symbols specific for each group (group A - triangle, group B - square and group NegCTL - circle). Mean values are given in the form of bar charts in the background and standard deviations as positive values above the bars. Mean values were analyzed for statistical differences with *P* ≤ 0.05 as indicated by lower case letters for comparing time points above the respective bars. As there was no significant difference (*P* > 0.20) between group A and B for any sampling point concerning the anti-rPA83 IgG antibody titers, letters were displayed across both groups. Mean values with identical letters were not significant different from each other. Means from group NegCTL were always under the detection limit and therefore not included in the letter display. FIS: Formalin inactivated spores; rBcLA: Recombinant *Bacillus* collagen-like antigen; rPA: Recombinant protective antigen

Titers of NegCTL group remained below the detection threshold (< 100) throughout the study. The F-tests indicated differences between time points, but titers of groups A and B show a similar development over time (*P* = 0.47) and means were not significantly different (*P* = 0.20). Mean anti-rPA83 titers increased significantly in both group A and B after the first and second vaccination (*P* = 0.0001) but decreased after the 3rd vaccination (*P* = 0.0051). Mean titers of goats surviving the challenge remained either nearly unchanged or raised strongly compared to values after second and third vaccination, leading to high variations within the groups (Fig. 2 and Additional file 1: Table S1).

### Toxin neutralization titer (NT_50_)

**Fig. 3:**
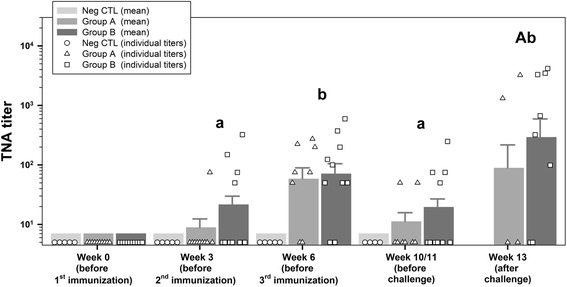
Toxin neutralization assay (TNA)-titers. Displayed are toxin-neutralizing titers for individual animals as well as predicted means and standard deviations for group A (rBclA + rPA83), B (rBclA + rPA83 + FIS) and NegCTL (unvaccinated negative control) on all sampling points. Values below the detection limit (<50) were given an arbitrary value of . Individual values are given as symbols specific for each group (group A - triangle, group B - square and group NegCTL - circle). Means are given in the form of bar charts in the background and standard deviations as positive values above the bars. Results were analyzed for statistical differences with *P* ≤ 0.05 indicated by lower case letters for comparing time points and upper case letters for comparing groups above the respective bars. Mean values of different time points with identical lower case letters were not significant different from each other. Mean values of different groups with identical upper case letters were not significant different from each other. As there was no significant difference (*P* > 0.25) between group A and B for any sampling point concerning the toxin-neutralizing titers, letters were displayed across both groups. Mean values with identical letters were not significant different from each other. Means from group NegCTL and before immunization were always under the detection limit and therefore not included in the letter display. FIS: Formalin inactivated spores; rBcLA: Recombinant *Bacillus* collagen-like antigen; rPA: Recombinant protective antigen

Sera of group NegCTL as well as sera of all goats before the first immunization remained below the detection limit (NT_50_ ≤ 50). The F-tests indicated differences between time points, but titers of groups A and B show a similar development over time (*P* = 0.81) and means were not significantly different (*P* = 0.25). After the first immunization only individual goats of groups A and B developed weak NT_50_ whereas the mean titers across groups A and B increased significantly (*p* = 0.0023) after the second immunization. In line with the tendency observed for anti-rPA83 IgG, the third immunization resulted in significant though lower mean titers (*P* = 0.0028) (Figs. 2 and 3, week 10/11). NT_50_ of goats surviving the challenge were highly variable (Fig. 3 and Additional file 1: Table S2). Animal showing high values for anti-rPA83 IgG also show high NT_50_ values (*r* = 0.956).

### Anti-rBclA-IgG titer

**Fig. 4:**
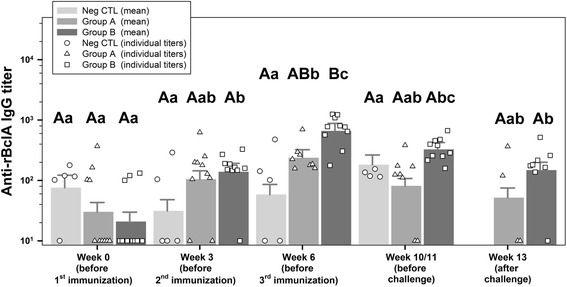
Anti-rBclA ELISA titers. Displayed are IgG antibody-values for individual animals as well as predicted means and standard deviations for group A (rBclA + rPA83), B (rBclA + rPA83 + FIS) and NegCTL (unvaccinated negative control) on all sampling points measured via indirect ELISA against rBclA. Values below the detection limit (<100) were given an arbitrary value of 10. Individual values are given as symbols specific for each group (group A - triangle, group B - square and group NegCTL - circle). Means are given in the form of bar charts in the background and standard deviations as positive values above the bars. Results were analyzed for statistical differences with *P* ≤ 0.05 indicated by lower case letters for comparing time points and upper case letters for comparing groups above the respective bars. Mean values of different time points with identical lower case letters were not significant different from each other. Mean values of different groups with identical upper case letters were not significant different from each other. Inter-group comparisons showed a significantly elevated mean IgG titer against rBclA for group B as compared to group NegCTL in week 6 only. FIS: Formalin inactivated spores; rBcLA: Recombinant *Bacillus* collagen-like antigen; rPA: Recombinant protective antigen

The F-tests indicated a significant interaction term, thus different development of titers over time in different groups. The mean titers of group B slightly increased after the first and second immunization (*P* = 0.0101 and *P* = 0.0078) but differ significantly only in week 6 from the negative control group (Fig. 4 and Additional file 1: Table S3).

### Anti-FIS-IgG titer

**Fig. 5:**
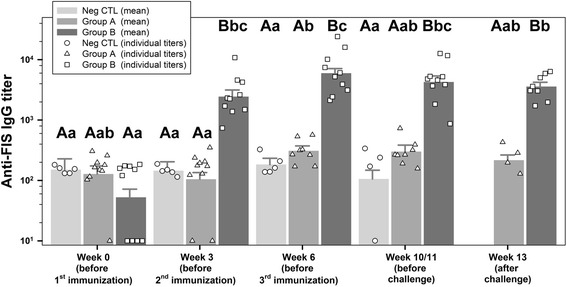
Anti-FIS (formalin inactivated spores) ELISA titers. Displayed are IgG antibody-values for individual animals as well as predicted means and their standard deviations for group A (rBclA + rPA83), B (rBclA + rPA83 + FIS) and NegCTL (unvaccinated negative control) on all sampling points measured via indirect ELISA against FIS (formalin inactivated spores). Values below the detection limit (<100) were given an arbitrary value of 10 for better display. Individual values are given as symbols specific for each group (group A - triangle, group B - square and group NegCTL - circle). Means are given in the form of bar charts in the background and standard deviations as positive values above the bars. Results were analyzed for statistical differences with *P* ≤ 0.05 indicated by lower case letters for comparing time points and upper case letters for comparing groups above the respective bars. Mean values of different time points with identical lower case letters were not significant different from each other. Mean values of different groups with identical upper case letters were not significant different from each other. Inter-group comparisons showed significantly elevated IgG titers for group B as compared to group A against FIS for all sampling points with the exception of week 0. FIS: Formalin inactivated spores; rBcLA: Recombinant *Bacillus* collagen-like antigen; rPA: Recombinant protective antigen

Again, F-tests indicated a significant interaction term, thus different development of titers over time in different groups. The mean titer of group B was significantly increased after the first (*P* < 0.0001) and second immunization (*P* = 0.0027) and stayed nearly constant after the third immunization and challenge (*P* = 0.1573, *P* = 0.4273). Titers from the NegCTL group were constant over time. Titers from group A, where FIS was not constituent of the vaccine formulation, remained nearly unchanged compared to serum before vaccination (Fig. 5 and Additional file 1: Table S4). Pooled group sera of every sampling point tested negative for IgM antibodies against rBclA and FIS (data not shown).

### Challenge

**Fig. 6:**
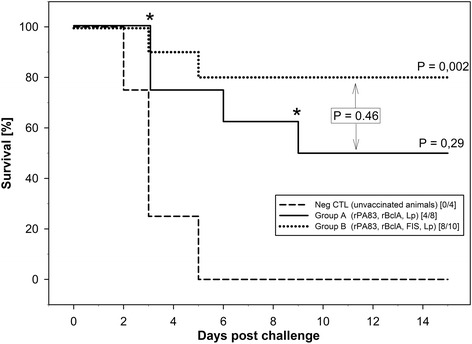
Survival data. Displayed are survival data of goats immunized with protein component vaccines with or without FIS (formalin inactivated spores) and challenged with fully virulent wild type strain *Bacillus anthracis* K-136. Survival was monitored up to 15 days. Significance was tested via a log-rank test. *P*-values are as compared to the unvaccinated animals if not indicated otherwise. The time of death was either the day the animal was found dead or the day of antibiotic treatment, as was the case for 2 goats of group A. The time points of their antibiotic treatment are labelled with * in the figure

In Fig. 6 the time to death curves are plotted, taking only full days into consideration. Two animals of group A got antibiotic treatment after a positive blood smear on days 3 and 9, respectively, according to the end point rules of this experiment. These animals were considered fatally infected and the day of intervention was taken for calculation of the death curve. Using the test for multiple comparisons group B shows a significant better survival than the negative control, with 8 out of 10 survivors (*P* = 0.0015) while group A with 4 out of 8 survivors did not (*P* = 0.2895). The difference in survival rate between group A and B is not significant (*P* = 0.4614) using the test approach described above. This test approach simultaneously used data from all goats. Note that using three two-sample log rank tests with a Bonferroni adjustment for multiple testing here showed significant increased survival rate for both groups A and B as compared to the unvaccinated negative group (NegCTL).

## Discussion

Living spore vaccines, currently used as one of the most important measures for controlling anthrax in animals, are not suitable for simultaneous antibiotic treatment and vaccination. This scenario is relevant in veterinary practice when vaccination is desirable to prevent the re-activation of an outbreak after the initial antibiotic treatment or when other non-living components are used in so-called combination vaccines to immunize simultaneously against both *B. anthracis* and a different pathogen.

A growing body of data from vaccinations of laboratory animals show the superiority of vaccines supposed to elicit protective immune responses against both the action of the toxins and the replication of the bacterium. We sought to know whether an appropriate non-living combination vaccine could protect one of the most susceptible species of livestock, goats, against a lethal challenge with *B. anthracis*. This study demonstrated that goats were protected by a combination of recombinant peptides with or without FIS from a lethal *B. anthracis* challenge.

Based on promising results from earlier vaccination experiments in mice [56], goats were immunized three times at 3-week intervals with rPA and rBclA without (group A) or plus *B. anthracis* 34F2 strain derived FIS (group B) adjuvanted by a lipopeptide. This robust vaccination regimen was chosen in an “all or nothing” approach to ensure that in this pioneering experiment even a minimal protectiveness would be verifiable. The challenge with ~1000 spores of a fully virulent strain of *B. anthracis* revealed 50% (4/8) and 80% (8/10) survival for the 14 days monitoring period in group A and B, respectively, while all animals (4/4) of the negative control died within 5 days. According to the Tukey test for multiple comparisons only group B showed a significant protection in comparison to the negative control. This result falls in line with the observations made earlier where a combination of rPA83 and different concentrations of FIS in guinea pigs and several mice strains resulted in full protection [39, 40] whereas the components given in single did not. ELISA and TNA titers measured after each vaccination indicate that a two-step vaccination could be sufficient for protection as titers were higher after second vaccination than after third (Figs. 2, 3). This needs to be further investigated to determine if a two-step vaccination will protect target animals as well and to compare the protectiveness in different livestock animals to those of the current Sterne live spore vaccine (SLSV). In a previous trial on the long-term protectiveness of the SLSV in goats [57] a 100% survival from lethal challenge was only achieved when goats were freshly boostered. The single application of SLSV resulted in 60% (6 weeks post vaccination) and 80% (62 weeks post vaccination) survival. While the protectiveness of the threefold vaccination schedule conducted here with the non-living vaccine looks comparable to a single vaccination with SLSV anti-PA and TNA-titers were equal or higher with the latter at 4–6 weeks after vaccination compared to the peak titers (week 6) with the non-living vaccine. However, the titers of both anti-PA and TNA were fairly comparable right before challenge. Interestingly, anti-FIS titers were much lower after a single vaccination with SLSV (<1/10) compared to our group B titers but increased comparably after a second vaccination with living spores. Taken in mind the role of an anti-spore immune response the high anti-FIS titers may have contributed to the higher survival seen in group B.

Some animals showed measurable, but low ELISA titers against rPA83, rBclA and FIS before immunization. This has been observed in other trials before [57] and can be attributed to unspecific reactivity, as the animals utilized in this study had no historical vaccination background and were not connected to any anthrax outbreak. Furthermore, the background titers of unvaccinated animals did not change significantly over time and conveyed no protection. Because of this we focused our statistical evaluation on the progressive change in titers over time and between groups clearly showing a vaccine effect in terms of seroconversion and survival in the vaccinated animals. Of note, two animals in group A and B showed no increase in anti-rPA and TNA titers after the survival of the challenge as compared to before the challenge hinting at a sterile immunity [58].

As the exosporium is the outermost surface of a *B. anthracis* spore, it has been the focus of numerous novel vaccination approaches by several laboratories. BclA forms the hair-like extensions protruding from the exosporium membrane [59, 60]. It has been shown to be immunodominant [61] and adds to protectiveness of PA based vaccines [33, 34, 56]. Goats injected with a protein formulation of rPA83, rBclA and lipopeptide (group A) showed little to no increase in ELISA titers using either rBclA or FIS as the antigen. Goats supplementary injected with FIS (group B) showed a strong increase in anti-FIS IgG titers but still an only marginal increase in titers against rBclA. Given the previously described results from mouse experiments the low responsiveness of goats to either the recombinant or FIS-derived BclA was unexpected. Interestingly, these results are in line with serological data from a study in goats vaccinated with the commercial Sterne spore vaccine. Vaccinates also showed no change in anti-rBclA IgG titers over the course of a year as compared to titers before the vaccination while anti-FIS IgG titers increased strongly after the first and again after a booster immunization with the Sterne spore vaccine [57]. It has been stated that the main antibody response against BclA is directed against the proteinaceous parts of the native protein [62] and several reports have shown the immunogenic and protective capacity of a non-glycosylated rBclA [32–34, 56]. However, we cannot completely rule out that goats in group B may have elicited antibodies against the carbohydrate moieties of the glycosylated FIS-derived BclA which were not detectable using rBclA as the antigen in our ELISA. If so, such antibodies might have added to the better protection in group B and immunogenic oligosaccharide side chains of BclA may be considered part of future non-living vaccines, too. Taken together these results support the previously described notion [27, 29] that BclA, albeit highly immunogenic, might not be the only or even main relevant antigen from *B. anthracis* spores. Vergis et al. [29] observed an even better survival rate in mice challenged with Ames spores after immunization with a ΔBclA strain of the anthracis-like *B. cereus* G9241. The authors explained these surprising results with observations by Cybulski et al. [36] and Basu et al. [63], the latter demonstrating that the removal of the exosporium from a spore enhances the cytokine response in macrophages while the exosporium itself is inert in this regard. More recently, Wang et al. [64] implicated BclA in impairing antibody responses and protection against lethal infection of mice through inhibition of complement. The results of our study indicate that BclA might not only partially mask recognition of important antigens beneath the exosporium, but is in addition poorly immunogenic in goats as compared to mice and rabbits. If this has implications concerning the high susceptibility of goats against an infection with *B. anthracis*, needs further investigation.

## Conclusions

The results of our study indicate the potential of an antigenic mixture eliciting an immune response against both the toxins and the spore components of *B. anthracis* to protect the extremely susceptible goat from a lethal infection with *B. anthracis*. The non-living nature of this vaccine would allow for a concomitant antibiotic treatment and vaccination procedure. Data from serological studies in goats support the robustness of immunogenicity of this vaccine also when administered in combination with long-term antibiotics (unpublished). Further studies will clarify how this vaccine candidate performs in a post infection scenario controlled by antibiotics.

## Additional file

Additional file 1: Supporting tables. **Table S1.** Anti-rPA83 IgG titers. **Table S2.** TNA titers. **Table S3.** Anti-rBclA IgG titers. **Table S4.** Anti-FIS IgG titers. (XLSX 24 kb)

## Abbreviations

Ah: Alhydrogel
AVA: Anthrax Vaccine Adsorbed
AVP: Anthrax Vaccine Precipitated
B: *Bacillus*
BclA: *Bacillus* collagen-like antigen
ELISA: Enzyme Linked Immunosorbent Assay
ET: Endotoxin
FIS: Formalin Inactivated Spores
FPLC: Fast protein liquid chromatography
HRP: Horseredish Peroxidase
IgG: Immunoglobulin G
IgM: Immunoglobulin M
IPTG: Isopropyl-*β*-D-thiogalactopyranosid
LF: Lethal Factor
LP: Lipopeptide
NegCTL: Negative Control group
NT: Neutralization Titer
PA: Protective Antigen
PBS: Phosphate Buffered Saline
PCR: Polymerase Chain Reaction
RBclA: Recombinant BclA
rPA: Recombinant Protective Antigen
SDS: Sodium Dodecyl Sulfoxid
SLSV: Sterne’s Live Spore Vaccine
TNA: Toxin Neutralization Assay

## References

[1] Pasteur L (1881). De l'atenuation des virus et de leur retour a la virulence. C Rend Acad Sci.

[2] Sterne M (1939). The immunization of laboratory animals against anthrax. Onderstepoort J Vet Sci Anim Indust.

[3] Turnbull PCB (1991). Anthrax vaccines: past, present and future. Vaccine.

[4] Turnbull PCB. Anthrax in Humans and Animals. WHO Press, 4th ed Geneva; 2008.

[5] Sterne M (1939). The use of anthrax vaccines prepared from avirulent (uncapsulated) variants of bacillus anthracis. Onderstepoort J Vet Sci Anim Indust..

[6] Sterne M, Nicol J, Lambrechts MC (1942). The effect of large-scale active immunisation against anthrax. J S Afr Vet Med Assoc.

[7] Little SF, Knudson GB (1986). Comparative efficacy of *Bacillus anthracis* live spore vaccine and protective antigen vaccine against anthrax in the guinea pig. Infect Immun.

[8] Turnbull PCB, Broster MG, Carman JA, Manchee RJ, Melling J (1986). Development of antibodies to protective antigen and lethal factor components of anthrax toxin in humans and guinea pigs and their relevance to protective immunity. Infect Immun.

[9] Ivins BE, Welkos SL, Knudson GB, Littl SF (1990). Immunization against anthrax with aromatic compound-dependent (Aro-) mutants of *Bacillus anthracis* and with recombinant strains of *Bacillus subtilis* that produce anthrax protective antigen. Infect Immun.

[10] Stepanov AV, Marinin LI, Pomerantsev AP, Staritsin NA (1996). Development of novel vaccines against anthrax in man. J Biotechnol.

[11] Webster A (1973). Inhibiting effect of antibiotics on anthrax vaccination. Aust Vet J.

[12] Ionin B (2013). Evaluation of Immunogenicity and Efficacy of Anthrax Vaccine Adsorbed for Postexposure Prophylaxis. Clin Vaccine Immunol.

[13] Cybulski RJ, Sanz P, O’Brien AD (2009). Anthrax vaccination strategies. JMAM.

[14] Baillie LWJ (2006). Past, imminent and future human medical countermeasures for anthrax. J Appl Microbiol.

[15] Scorpio A, Blank TE, Day WA, Chabot DJ (2006). Anthrax vaccines: Pasteur to the present. Cell Mol Life Sci.

[16] Little SF (2005). Anthrax vaccines. BioDrugs.

[17] Turnbull PCB, Artenstein AW (2010). Anthrax vaccines. Vaccines: a biography.

[18] Welkos SL, Friedlander AM (1988). Comparative safety and efficacy against *Bacillus anthracis* of protective antigen and live vaccines in mice. Microb Pathog.

[19] Ivins BE, Welkos SL, Little SF, Crumrine MH, Nelson GO (1992). Immunization against anthrax with *Bacillus anthracis* protective antigen combined with adjuvants. Infect Immun.

[20] Ivins B, Fellows PF, Pitt ML, Estep J, Farchaus JW, Friedlander AM, Gibbs PH (1995). Experimental anthrax vaccines: efficacy of adjuvants combined with protective antigen against an aerosol *Bacillus anthracis* spore challenge in guinea pigs. Vaccine.

[21] Ivins BE, Pitt ML, Fellows PF, Farchaus JW, Benner GE, Waag DM, Little SF, Anderson GW, Gibbs PH, Friedlander AM (1998). Comparative efficacy of experimental anthrax vaccine candidates against inhalation anthrax in rhesus macaques. Vaccine.

[22] Fellows PF, Linscott MK, Ivins BE, Pitt ML, Rossi CA, Gibbs PH, Friedlander AM (2001). Efficacy of a human anthrax vaccine in guinea pigs, rabbits, and rhesus macaques against challenge by *Bacillus anthracis* isolates of diverse geographical origin. Vaccine.

[23] Ivins BE, Ezzell JW, Jemski J, Hedlund KW, Ristroph JD, Leppla SH (1986). Immunization studies with attenuated strains of bacillus anthracis. Infect Immun.

[24] Welkos SL, Friedlander AM (1988). Pathogenesis and genetic control of resistance to the Sterne strain of bacillus anthracis. Microb Pathog.

[25] Cohen S, Mendelson I, Altboum Z, Kobiler D, Elhanany E, Bino T, Leitner M, Inbar I, Rosenberg H, Gozes Y, Barak R, Fisher M, Kronman C, Velan B, Shafferman A (2000). Attenuated nontoxinogenic and nonencapsulated recombinant *Bacillus anthracis* spore vaccines protect against anthrax. Infect Immun.

[26] Rhie GE, Roehrl MH, Mourez M, Collier RJ, Mekalanos JJ, Wang JY (2003). A dually active anthrax vaccine that confers protection against both bacilli and toxins. Proc Natl Acad Sci U S A.

[27] Cote CK, Kaatz L, Reinhardt J, Bozue J, Tobery SA, Bassett AD, Sanz P, Darnell SC, Alem F, O’Brien AD, Welkos SL (2012). Characterization of a multi-component anthrax vaccine designed to target the initial stages of infections well as toxaemia. J Med Microbiol.

[28] Uchida M, Harada T, Enkhtuya J, Kusumoto A, Kobayashi Y, Chiba S, Shyaka A, Kawamoto K (2012). Pro-tective effect of bacillus anthracis surface protein EA1 against anthrax in mice. Biochem Biophy Res Commun.

[29] Vergis JM, Cote CK, Bozue J, Alem F, Ventura CL, Susan L, Welkos SL, O’Briena AD (2013). Immunization of mice with formalin-inactivatedspores from avirulent Bacillus Cereus strains provides significant protection from challenge with bacillus anthracis Ames. Clin Vaccine Immunol.

[30] Klein NF, DeArmon IA, Lincoln RE, Mahlnadt BG, Fernelius AL (1962). Immunological studies of anthrax. II. Levels of immunity against bacillus anthracis obtained with protective antigen and live vaccine. J Immunol.

[31] Brossier F, Weber-Levy M, Mock M, Sirard JC (2000). Role of toxin functional domains in anthrax pathogenesis. Infect Immun.

[32] Ivins BE, Welkos S (1988). Recent advances in the development of an improved humen anthrax vaccine. Eur J Epidemiol.

[33] Brahmbhatt TN, Darnell SC, Carvalho HM, Sanz P, Kang TJ, Bull RL, Rasmussen SB, Cross AS, O'Brien AD (2007). Recombinant exosporium protein BclA of bacillus anthracis is effective as a booster for mice primed with suboptimal amounts of protective antigen. Infect Immun.

[34] Hahn UK, Böhm R, Beyer W (2006). DNA vaccination against anthrax in mice – combination of anti-spore and anti-toxin components. Vaccine.

[35] Cote CK, Bozue J, Moody KL, DiMezzo TL, Chapman CE, Welkos SL (2008). Analysis of a novel spore antigen in bacillus anthracis that contributes to spore opsonization. Microbiology.

[36] Cybulski RJ, Sanz P, McDaniel D, Darnell S, Bull RL, O’Brien AD (2008). Recombinant bacillus anthracis spore proteins enhance protection of mice primed with suboptimal amounts of protective antigen. Vaccine.

[37] Enkhtuya J, Kawamoto K, Kobayashi Y, Uchida I, Rana N, Makino S (2006). Significant passive protective effect against anthrax by antibody to bacillus anthracis inactivated spores that lack two virulence plasmids. Microbiology.

[38] Baillie LWJ, Hebdon R, Flick-Smith H, Williamson D (2003). Characterisation of the immune response to the UK human anthrax vaccine. FEMS Immunol Med Microbiol.

[39] Brossier F, Levy M, Mock M (2002). Anthrax spores make an essential contribution to vaccine efficacy. Infect Immun.

[40] Gauthier YP, Tournier JN, Paucod JC, Corre JP, Mock M, Goossens PL, Vidal DR (2009). Efficacy of a vaccine based on protective antigen and killed spores against experimental inhalational anthrax. Infect Immun.

[41] Glomski IJ, Corre JP, Mock M, Goossens PL (2007). Cutting edge: IFN-c-producing CD4 T lymphocytes mediate spore induced immunity to capsulated bacillus anthracis. J Immunol.

[42] Loleit M, Ihlenfeldt HG, Brünjes J, Jung G, Müller B, Hoffmann P, Bessler WG, Pierres M, Haas G (1996). Synthetic peptides coupled to the lipotripeptide P3CSS induce in vivo B and T helper cell responses to HIV-1 reverse transcriptase. Immunobiology.

[43] Schild H, Deres K, Wiesmüller KH, Jung G, Rammensee HG (1991). Efficiency of peptides and lipopeptides for in vivo priming of virus-specific cytotoxic T cells. Eur J Immunol.

[44] Schlecht S, Wiesmüller K-H, Jung G, Bessler WG (1993). Lipopeptide als natürliche Adjuvantien für Impfstoffe aus Gram-negativen Bakterien. Naturwissenschaften.

[45] Wiesmüller KH, Jung G, Hess G (1989). Novel low-molecular- weight synthetic vaccine against foot-and-mouth disease containing a potent B-cell and macrophage activator. Vaccine.

[46] Wiesmüller KH, Fleckenstein B, Jung G (2001). Peptide Vaccines and Peptide Libraries. Biol Chem.

[47] Mittenbühler K, vdEsche U, Heinevetter L, Bessler WG, Huber M (2003). Lipopeptides: adjuvanticity in conventional and genetic immunization. FEMS Immunol Med Microbiol.

[48] Beyer W, Hahn UK, Boehm R. Lipopeptide adjuvant versus aluminium hydroxide in PA based vaccines. 5^th^ International BACT-Conference. Abstract book. 2005;P14:49.

[49] Gladstone GP (1946). Immunity to anthrax: protective antigen present in cell-free culture filtrates. Br J Exp Pathol.

[50] Schlingman AS, Devlin HB, Wright GG, Maine RJ, Manning MC (1956). Immunizing activity of alum-precipitated protective antigen of bacillus anthracis in cattle, sheep, and swine. Am J Vet Res.

[51] Jackson FC, Wright GG, Armstrong J (1957). Immunization of cattle against experimental anthrax with alum-precipitated protective antigen or spore vaccine. Am J Vet Res.

[52] Ghielmetti M, Reschner A, Zwicker M, Padovan E (2005). Synthetic bacterial lipopeptide analogs: structural requirements for adjuvanticity. Immunbiol.

[53] Hahn UK, Alex M, Czerny CP, Boehm R, Beyer W (2004). Protection of mice against challenge with bacillus anthracis STI spores after DNA vaccination. Int J Med Microbiol.

[54] Buyuk F, Sahin M, Cooper C, Celebi O, Saglam AG, Baillie L, Celik E, Akca D, Otluet S. The effect of prolonged storage on the virulence of isolates of Bacillus anthracis obtained from environmental and animal sources in the Kars Region of Turkey. FEMS Microbiol Lett. 2015;362:fnv102. 10.1093/femsle/fnv102 26109359

[55] Joellenbeck LM, Zwanziger LL, Durch JS, Strom BL (2002). The Anthrax Vaccine: Is it safe? Does it work? In: Committee to Assess the Safety and Efficacy of the Anthrax Vaccine. Medical Follow-Up Agency, Institute of Medicine.

[56] Köhler SM, Baillie LW, Beyer W (2015). BclA and toxin antigens augment each other to protect NMRI mice from lethal bacillus anthracis challenge. Vaccine.

[57] Ndumnego OC, Köhler S, Crafford J, van Heerden H, Beyer W (2016). Comparative analysis of the immunologic response and protective efficacy induced by the Sterne 34F2 live spore *Bacillus anthracis* vaccine in a ruminant model. Vet Immunol Immunop.

[58] Hermanson G (2004). A cationic lipid-formulated plasmid DNA vaccine confers sustained antibody-mediated protection against aerosolized anthrax spores. Proc Natl Acad Sci U S A.

[59] Sylvestre P, Couture-Tosi E, Mock M (2002). A collagen-like surface glycoprotein is a structural component of the bacillus anthracis exosporium. Mol Microbiol.

[60] Sylvestre P, Couture-Tosi E, Mock M (2003). Polymorphism in the collagen-like region of the bacillus anthracis BclA protein leads to variation in Exosporium filament length. J Bacteriol.

[61] Steichen CT, Kearney JF, Turnbough CL (2005). Characterization of the exosporium basal layer protein BxpB of bacillus anthracis. J Bacteriol.

[62] Steichen C, Chen P, Kearney JF, Turnbough CL (2003). Identification of the immunodominant protein and other proteins of the bacillus anthracis exosporium. J Bacteriol.

[63] Basu S, Kang TJ, Chen WH, Fenton MJ, Baillie L, Hibbs S, Cross AS (2007). Role of bacillus anthracis spore structures in macrophage cytokine responses. Infect Immun.

[64] Wang Y, Jenkins SA, Gu C, Shree A, Martinez-Moczygemba M, Herold J, Botto M, Wetsel RA, Xu Y (2016). Bacillus anthracis spore surface protein BclA mediates complement factor H binding to spores and promotes spore persistence. PLoS Pathog.

